# Unconventional PDZ Recognition Revealed in α7 nAChR-PICK1 Complexes

**DOI:** 10.1021/acschemneuro.4c00138

**Published:** 2024-05-01

**Authors:** Vasyl Bondarenko, Qiang Chen, Tommy S. Tillman, Yan Xu, Pei Tang

**Affiliations:** † Depatment of Anesthesiology and Perioperative Medicine, University of Pittsburgh, Pittsburgh, Pennsylvania 15260, United States; ‡ Department of Structural Biology, University of Pittsburgh, Pittsburgh, Pennsylvania 15260, United States; § Department of Pharmacology and Chemical Biology, University of Pittsburgh, Pittsburgh, Pennsylvania 15260, United States; ∥ Department of Physics and Astronomy, University of Pittsburgh, Pittsburgh, Pennsylvania 15260, United States; ⊥ Department of Computational and Systems Biology, University of Pittsburgh, Pittsburgh, Pennsylvania 15260, United States

**Keywords:** PDZ, PICK1, α7 nicotinic acetylcholine receptor, α7 nAChR, NMR

## Abstract

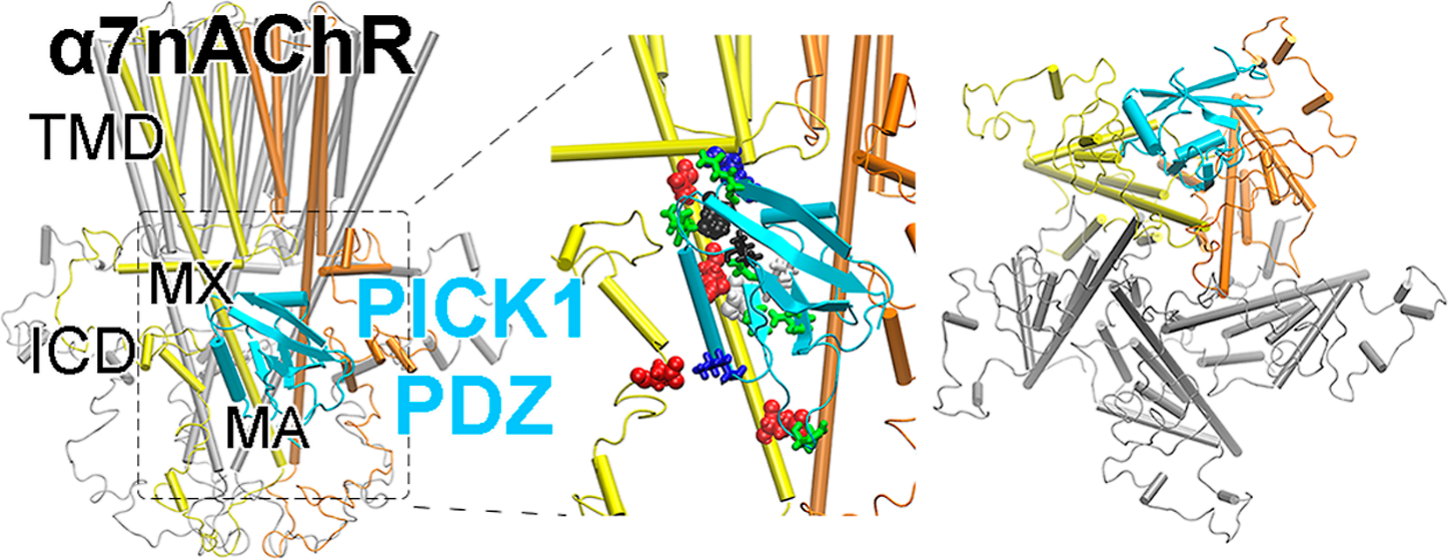

PDZ domains are modular domains that conventionally bind to C terminal or internal motifs of target proteins to control cellular functions through the regulation of protein complex assemblies. Almost all reported structures of PDZ-target protein complexes rely on fragments or peptides as target proteins. No intact target protein complexed with PDZ was structurally characterized. In this study, we used NMR spectroscopy and other biochemistry and biophysics tools to uncover insights into structural coupling between the PDZ domain of protein interacting with C-kinase 1 (PICK1) and α7 nicotinic acetylcholine receptors (α7 nAChR). Notably, the intracellular domains of both α7 nAChR and PICK1 PDZ exhibit a high degree of plasticity in their coupling. Specifically, the MA helix of α7 nAChR interacts with residues lining the canonical binding site of the PICK1 PDZ, while flexible loops also engage in protein–protein interactions. Both hydrophobic and electrostatic interactions mediate the coupling. Overall, the resulting structure of the α7 nAChR-PICK1 complex reveals an unconventional PDZ binding mode, significantly expanding the repertoire of functionally important PDZ interactions.

## Introduction

The post-synaptic density-95, disks-large, and zonula occludens-1 (PDZ) domain is a structurally well-conserved domain found in many proteins that are otherwise unrelated. It serves as one of the most common mediators for establishing protein complexes,^1,2^ which regulate a broad range of functions, including cell signaling, synaptic efficacy and plasticity, cell–cell junctions, and cellular trafficking and surface retention of ion channels.^3,4^ Dysregulation of PDZ domain function has been linked to the onset of various pathologies.^5^ As a result, PDZ domains have been the subject of intensive research and are considered a promising target for pharmaceutical interventions.^5,6^

**Figure 1:**
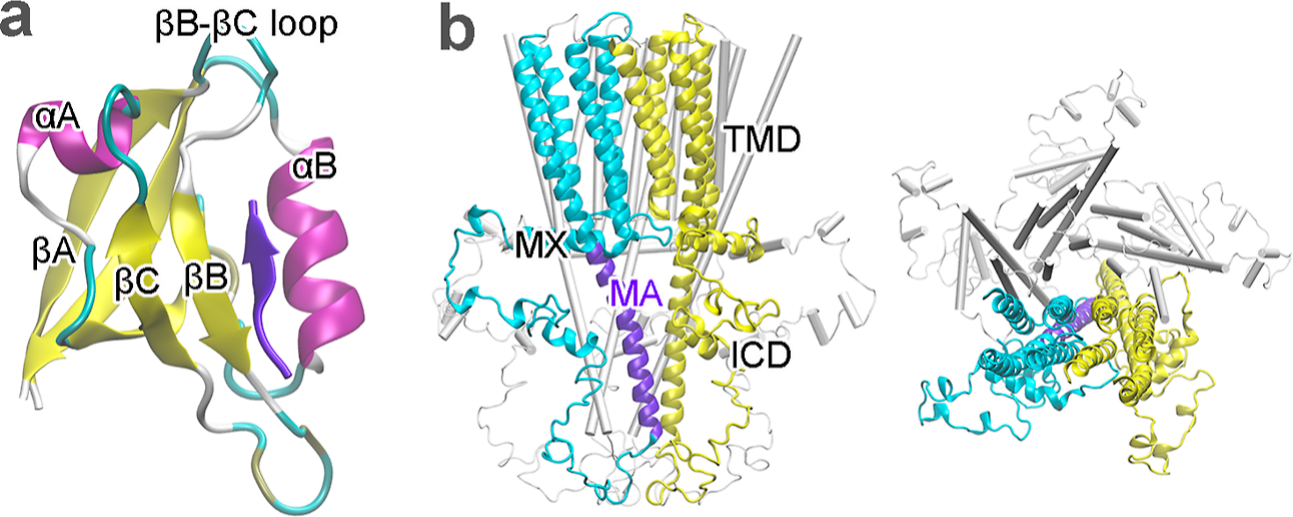
Structures of the PICK1 PDZ domain and the α7 nAChR transmembrane and intracellular domains (TMD + ICD). (a) Representative PDZ domain of PICK1 (PDB code: 2PKU). A bound peptide (violet) highlights the canonical binding groove consisting of the βB-βC loop, βB strand, and αB helix. (b) Side (left) and top (right) views of a representative pentameric structure of the α7 nAChR TMD + ICD (PDB code: 7RPM). The MA and MX helices in the ICD are marked.

Protein interacting with C kinase-1 (PICK1) is a PDZ-containing protein, which is abundant in the brain.^7^ Functionally, PICK1 regulates the trafficking and clustering of various membrane proteins, such as AMPA receptors,^8−11^ dopamine transporters,^12,13^ and α7 nicotinic acetylcholine receptors (α7 nAChR).^14−16^ PICK1 is implicated in chronic pain,^17−19^ addiction,^20^ Alzheimer’s disease,^21^ and other diseases and disorders.^22,23^ Structurally, PICK1 contains an amino-terminal PDZ domain that mediates PICK1 interactions with protein partners, particularly through the PDZ canonical binding groove (Figure 1a) that typically recognizes the extreme C-termini of target proteins.^24,25^ The recognized PDZ-binding sequence motifs include class I (X(S/T)Xφ) and class II (XφXφ),^8,12,13^ where X is any residue and φ is a hydrophobic residue. These conventional PDZ-binding motifs are mostly an extended β strand or coiled form.^2,26,27^ However, mutagenesis studies suggest that PICK1 binding to α7 nAChR may not follow class I and II consensus motifs but is likely involved with the canonical binding site of PICK1.^14^ PICK1 negatively regulates α7 nAChR surface clustering on hippocampal interneurons through its PDZ coupling with the MA helix in the intracellular domain (ICD) of α7 nAChR (Figure 1b).^14^ The structural basis underlying the α7 nAChR-PICK1 interactions remains a mystery.

α7 nAChR forms homopentameric ligand-gated ion channels (Figure 1b) and is expressed widely across the human body in both neuronal and non-neuronal cells.^28−31^ α7 nAChR mediates synaptic transmission in the central and peripheral nervous systems and is involved in cognitive function, mental health, and neurodegenerative diseases.^28^ α7 nAChR is also an important player in the cholinergic anti-inflammatory pathway.^29,32,33^ Deficiency of functional α7 nAChR in the hippocampus and other brain regions has been found to be associated with cognitive impairments and sensory processing deficits.^34,35^ The reduction of functional α7 nAChR can result from changes in α7 nAChR interactions with intracellular proteins, particularly those directly affecting α7 nAChR assembly and trafficking to and from the cell surface.^14,36,37^ However, there is little structural information about how α7 nAChR interacts with intracellular proteins, including α7 nAChR interactions with the PICK1 PDZ domain leading to suppression of surface α7 nAChR clustering.^14^ In fact, for other PICK1 interacting receptors, all complex structures of the PDZ domain determined previously show only bound peptides of the receptor tail without the structural context of intact receptors.^24,25,38,39^

Here, we have revealed structural insights into α7 nAChR binding to the PICK1 PDZ domain by carrying out NMR, mutagenesis, and other experiments. Guided by experimental restraints and available structures of α7 nAChR^16,40−42^ and PICK1,^24,25,38,39^ the generated α7 nAChR-PICK1 complex structures offer a new perspective in PDZ recognition of unconventional binding motifs, in particular helices in intact receptors. The finding opens a new path toward an understanding of protein–protein interactions and PDZ-regulated functions.

## Results

### Regions of α7 nAChR Critical for Binding PICK1

**Figure 2:**
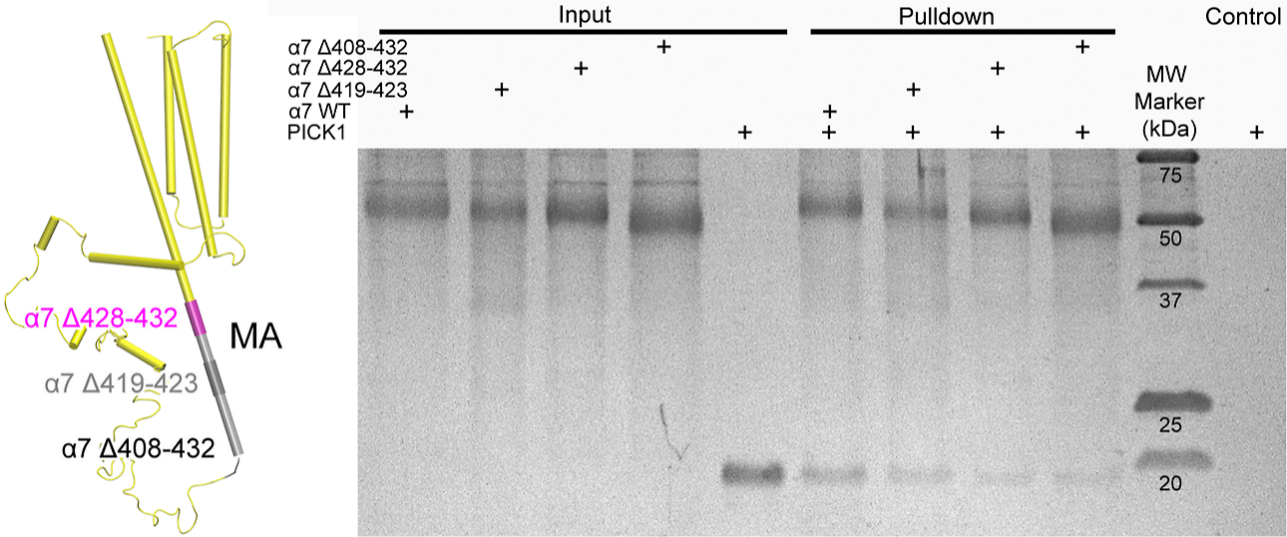
Critical role of the α7 nAChR MA helix in binding PICK1. Left: three α7 nAChR mutants with varied segment deletion of the MA helix as shown in a monomer of the α7 nAChR TMD + ICD structure; right: SDS-PAGE gel (15%) showing PICK1 (the PDZ domain) pulldown by the WT or mutants of his-tag α7 nAChR as baits. The purified PICK1 and α7 nAChR (WT or mutants) were mixed in a molar ratio of 5:1 and bound to NiNTA resin, which was incubated at 4 °C overnight and washed two times with 50 mM imidazole before elution with 0.5 M imidazole. The same procedure for sample incubation, washing, and elution was applied to the control sample (PICK1 only, without α7 nAChR) that resulted in no nonspecific PICK1 binding to the resin. The impact of MA helix deletions was also evaluated based on band intensity ratios (PICK1/α7 nAChR), as shown in Supporting Information, Figure S1.

The MA helix of α7 nAChR was suggested to be necessary and sufficient for binding the PICK1 PDZ domain, but the regions of the MA helix critical for the binding remain unknown.^14^ To determine the MA regions responsible for binding PICK1, we deleted different segments of the MA helix, obtained purified proteins of these α7 nAChR mutants, and tested their pulldown of the PICK1 PDZ domain (Figure 2). Deletion of a helical turn in the middle of the MA helix (α7Δ419–423) weakened the PICK1 pulldown by ∼50% compared to wild-type (WT) α7 nAChR; deletion of an upper helical turn (α7Δ428–432) had an even more profound effect and resulted in ∼85% reduction of the pulldown (Figure 2 and Supporting Information, Figure S1). Interestingly, mutant α7Δ408–432 showed almost the same reduction of PICK1 pulldown as α7Δ428–432 (Figure 2 and Supporting Information, Figure S1), suggesting a more important role of the upper than lower MA helix in binding PICK1.

### α7 nAChR Residues Involved in Binding PICK1

**Figure 3:**
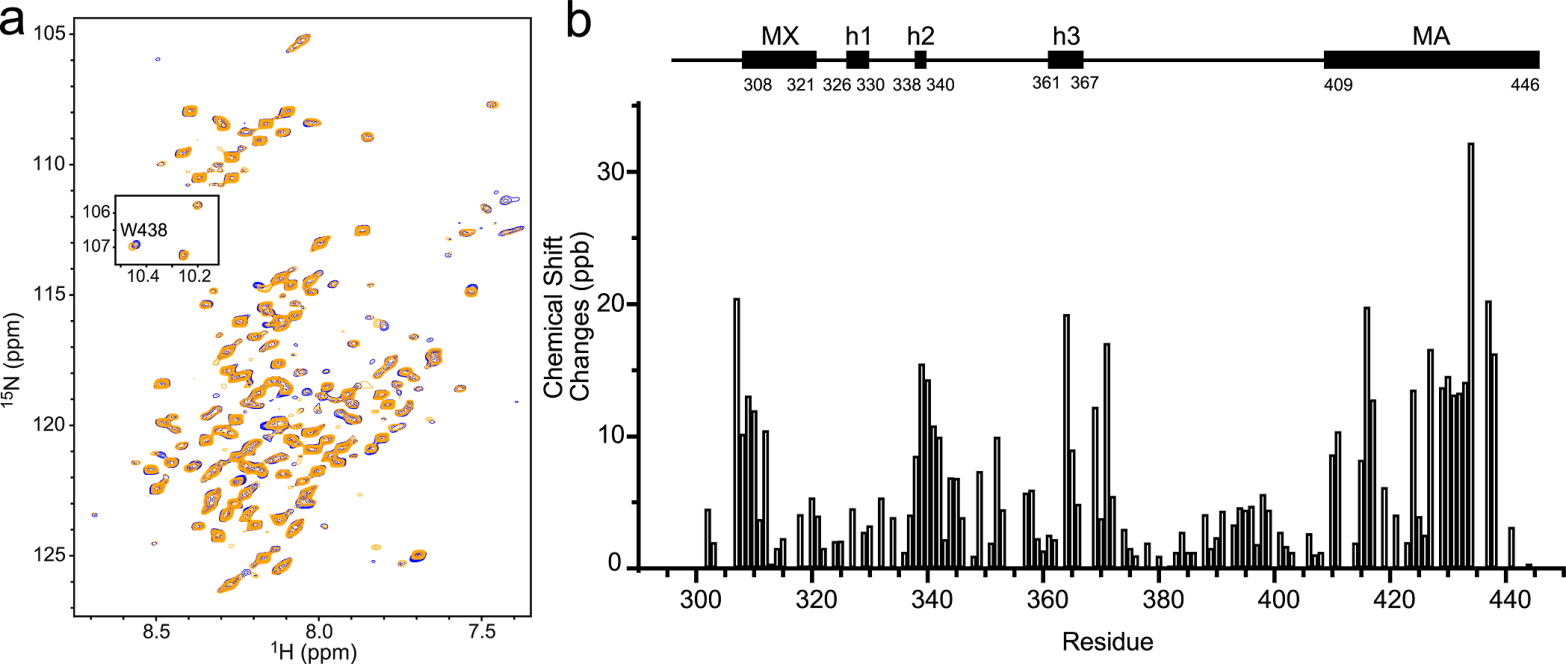
α7 nAChR CSPs induced by binding of the PICK1 PDZ domain. (a) Overlay of representative ^1^H–^15^N TROSY-HSQC NMR spectra of α7 nAChR TMD + ICD in the absence (blue) and presence (orange) of PICK1. Inset shows chemical shift changes of the W438 indole amide due to the addition of PICK1. NMR spectra with different concentrations of PICK1 are provided in Supporting Information, Figure S2. (b) α7 chemical shift changes (Δδ_HN_ = [Δδ_H_^2^ + Δδ_N_^2^/25]^1/2^) induced by PICK1 (210 μΜ). The spectra in (a) were collected on an 18.8 T NMR spectrometer at 45 °C. Missing bars in (b) are due to proline residues, overlapping residues, or weak NMR signals. Source data are provided as a Source Data file.

**Figure 4:**
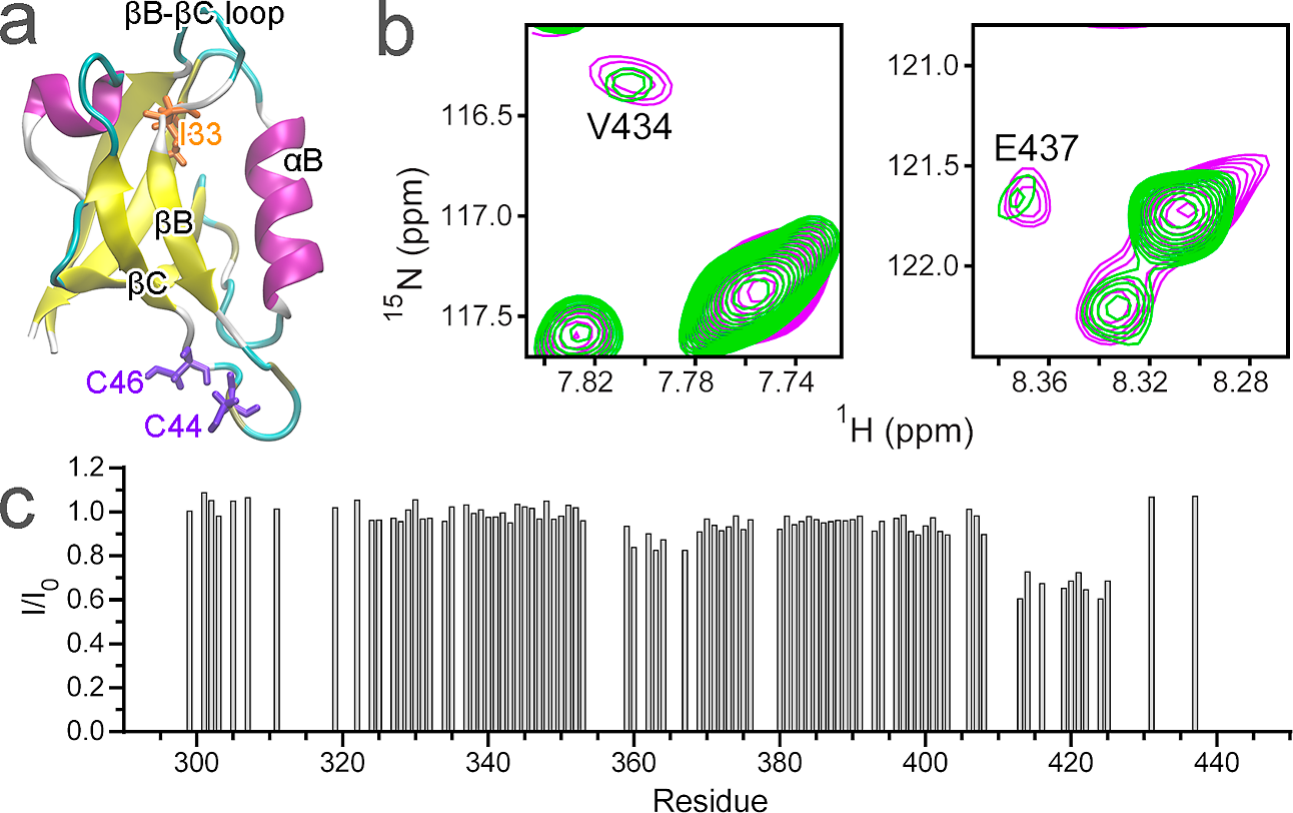
Additional NMR experiments for defining the structural relationship between α7 nAChR and PICK1. (a) Structure of the PICK1 PDZ domain showing residues that were used in NMR experiments for defining the structural interrelationship between α7 nAChR and PICK1. (b) Representative zoomed-in regions of the 2D saturation transfer (STD) NMR spectra of the α7 nAChR TMD + ICD without (purple) and with (green) saturation of the PICK1 I33 amide proton (∼11.6 ppm. See Figure a). Residues displaying profound intensity changes, such as V434 and E437, are <6 Å from I33. (c) Quantified changes of normalized PRE (*I*/*I*_0_) of the α7 nAChR ICD that were caused by the paramagnetic MTSL labeled to C44 and C46 of PICK1 as shown in (a). Missing bars in (c) are due to proline residues, overlapping residues, or weak NMR signals. Source data are provided as a Source Data file. The full NMR spectra for (b) and (c) are shown in Supporting Information, Figures S3 and S4.

To identify residues of α7 nAChR involved in binding PICK1, we used the α7 nAChR construct comprising the TMD + ICD^16,40^ for three lines of NMR experiments, including chemical shift perturbation (CSP) (Figure 3), paramagnetic relaxation enhancement (PRE) resulting from PICK1 labeled with MTSL (Figure 4), and two-dimensional saturation transfer difference (STD) NMR (Figure 4).^43,44^ In CSP experiments, ^1^H–^15^N heteronuclear single quantum coherence (HSQC) NMR spectra of α7 nAChR TMD + ICD were collected in the absence and presence of the unlabeled PICK1 PDZ protein. Residues showing CSP are potentially involved in binding PICK1 (Figure 3 and Supporting Information, Figure S2). Notably, in addition to the MA helical residues, some α7 nAChR residues in the MX helix and flexible loop also display CSP. These residues are facing away from the α7 nAChR channel and are accessible by PICK1. To further observe interaction details between two proteins, we performed intermolecular STD experiments.^43,44^ Residue I33 (Figure 4a) in the carboxylate-binding loop region of the PICK1 PDZ domain has a unique ^1^HN chemical shift at ∼11.6 ppm, where there are no other resonance signals. Cross-saturation from PICK1 (I33, ^1^HN) to α7 nAChR resulted in a profound decrease in the peak intensity of residues at the top of the α7 nAChR MA helix (Figure 4b), indicating the closeness of PICK1 I33 with those α7 nAChR residues, such as V434 and E437. A full spectrum of 2D STD is shown in Supporting Information, Figure S3. We also utilized two native cysteine residues (C44, C46; Figure 4a) of PICK1 for labeling the paramagnetic probe MTSL. Intermolecular PRE NMR experiments^16,40,45^ demonstrated reduced peak intensity for certain α7 nAChR residues, specifically residues 413–416 and 419–425 at the lower half of the MA helix (Figure 4c and Supporting Information, Figure S4). The PRE data indicate that residues C44 and C46 of PICK1 are closer to the lower half (residue number <426) than the upper half (residue number ≥426) of the α7 nAChR MA helix. Notably, these residues also exhibit CSP upon binding of PICK1 (Figure 3). Collectively, these findings elucidate the α7 nAChR residues involved in PICK1 binding and define their relative spatial arrangement.

### Binding Mode of the PICK1 PDZ Domain

**Figure 5:**
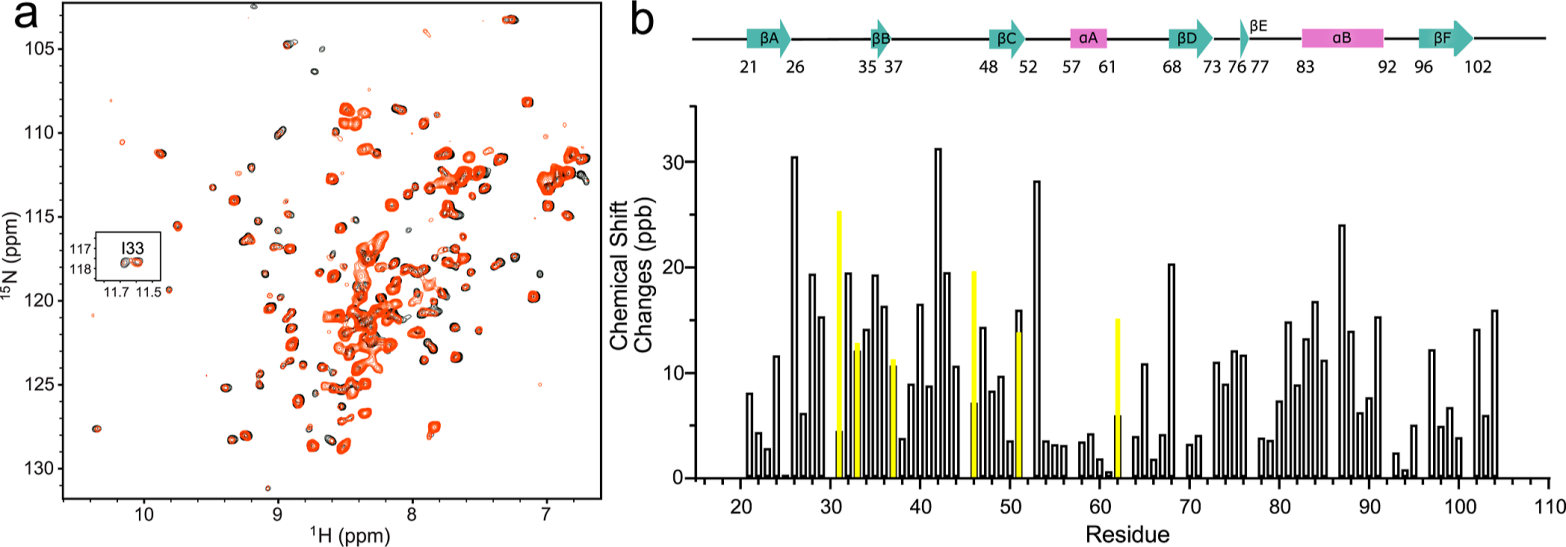
PICK1 CSPs induced by α7 nAChR. (a) Overlay of representative ^1^H–^15^N TROSY-HSQC NMR spectra of PICK1 in the absence (black) and presence (red) of α7 nAChR TMD + ICD. (b) PICK1 chemical shift changes induced by the α7 TMD + ICD (10 μΜ). Residues with yellow bars have two peaks, indicating a slow exchange of two conformations. The spectra in (a) were collected on an 18.8 T NMR spectrometer at 45 °C. Missing bars in (b) are due to proline residues, overlapping residues, or weak NMR signals. Source data are provided as a Source Data file.

A groove between the PDZ βB-strand and αB-helix (Figure 1a) is the canonical binding site for ligands or proteins.^46^ Does this canonical site also serve for binding α7 nAChR? The intermolecular STD experiment supports such a possibility, showing interactions between residue I33 at the PDZ canonical binding site and residues on the MA helix of α7 nAChR (Figure 4b). To obtain a more comprehensive picture, we collected ^1^H–^15^N HSQC NMR spectra of PICK1 in the absence and presence of unlabeled α7 nAChR (Figure 5a). Residues preceding or within the βB strand, including L32, I33, G34, I35, S36, and I37, show substantial CSP (Figure 5b). Residues near or on the αB helix (K81, V84, A87, Q91) as well as residues in loops connecting βB-βC (Q42, Y43) and βC-αA (Q51, F53) also show sizable CSP. These NMR data suggest that, in addition to the canonical binding site, some loop regions of the PICK1 PDZ domain are also involved in binding α7 nAChR. Furthermore, we performed pulldown experiments with FSC231, a specific PICK1 inhibitor that binds to the canonical groove of the PICK1 PDZ domain.^47,48^ FSC231 inhibits α7 nAChR-PICK1 pulldown in a concentration-dependent manner (Supporting Information, Figure S5). These results, along with the intermolecular PRE data (Figure 4c), were used to define the binding mode of the PICK PDZ domain and establish structural restraints for the α7 nAChR-PICK1 complexes.

### Structural Features of α7 nAChR-PICK1 Complexes

**Figure 6:**
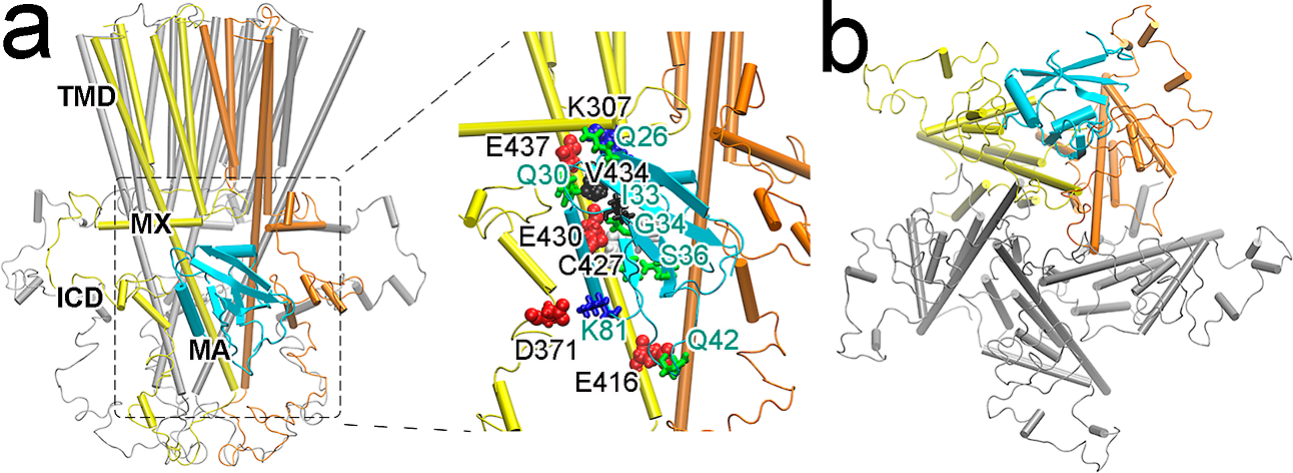
Structure models of α7 nAChR and PICK1 complexes, generated by HADDOCK based on NMR experimental restraints. (a) Side and (b) bottom views of a representative structure (PDBDEV_00000230) of α7 nAChR in complex with the PICK1 PDZ domain. A zoomed-in picture in (a) highlights interacting residues of α7 nAChR (presented in VDW) and PICK1 (presented in licorice) that are accountable for binding of the two proteins. Individual NMR structures of the α7 nAChR TMD + ICD (PDB code: 7rpm) and the PICK1 PDZ domain (PDB code: 2lui) were used for initiating the complex structure calculations. The quality of structural models is reported in Supporting Information, Table S2.

Guided by experimental restraints (Figures 2–5), we generated α7 nAChR-PICK1 structural models using HADDOCK^49,50^ (Supporting Information, Table S1). The quality of the final complex model was examined (Supporting Information, Table S2). Several structural features are notable (Figure 6). The PICK1 PDZ domain occupies the intersubunit space between two adjacent ICDs of α7 nAChR, where the MA helix on the principal side binds the PDZ canonical site and the α7 subunit on the complementary side mostly interacts with nearby PDZ loop residues. α7 nAChR residues facing away from the channel at the upper and middle MA helix (E437, V434, S431, E430, C427, N423) are in close contact with PICK1 residues near or on the βB strand (Q30, L32, G34, S36) (Figure 6 and Supporting Information, Figure S6). In contrast to α7 nAChR, the α4 and β2 nAChR subunits do not bind to the PICK1 PDZ domain^14^ despite sharing sequence similarities in their MA helices (as shown in Supporting Information, Figure S7). To validate the α7 nAChR-PICK1 structural model and assess the importance of the α7 MA helix in PICK1 binding, we introduced specific mutations into the α7 MA helix (S431D, E430D, and N423D) to mimic equivalent residues found in α4 or β2 nAChR (as depicted in Supporting Information, Figure S7). Remarkably, the mutant exhibited a significant reduction in PICK1 binding compared to the WT α7 nAChR in pull-down experiments (Supporting Information, Figure S7). The residual binding of PICK1 by the α7 mutant agrees with our structural model, which suggests that, in addition to the MA helix, the MX helix (K307 and R310) and the flexible loop (such as Q339, R340, Y364, and D371) also interact with the PICK1 PDZ domain (Supporting Information, Figure S6). The formation of a salt bridge between R310 of α7 nAChR and D28 of the PDZ domain (Supporting Information, Figure S6b) agrees with the previous mutagenesis finding^14^ in which D28A (along with K27A) abolished the PICK1 PDZ modulation to α7 nAChR clustering at the surface of GABAergic interneurons.^14^ Residues near (K81) or on (Q91) the PDZ helix αB contact the α7 nAChR ICD loop residues and even form a salt bridge (K81-D371) (Supporting Information, Figure S6d). These interactions at multiple sites jointly contribute to the binding of the two proteins.

## Discussion

PDZ-mediated protein interactions play a crucial role in the regulation of a wide array of biological processes. Understanding the structural aspects of PDZ binding is essential for both mechanistic insights and therapeutic advancements. Notably, most published PDZ complex structures involve peptides derived solely from the extreme C-termini of target proteins.^51^ Those peptide-PDZ complex structures suffice for certain proteins, such as AMPA receptors^8−11^ and dopamine transporters,^12,13^ where the unstructured C-terminal ends do not closely interact with other regions of the proteins. However, the α7 nAChR-PICK1 PDZ interaction represents a significantly more intricate partnership. In the study presented here, the structural model of the α7 nAChR-PICK1 PDZ complex provides, for the first time, a comprehensive view of how a PDZ domain interacts with an intact domain of a channel protein.

Several structural features are noteworthy. First, the MA helix of α7 nAChR interacts with the PDZ canonical binding groove of PICK1. The molecular interaction pattern of the PDZ for the MA helix does not match patterns for extended peptide motifs,^24^ demonstrating the flexibility of the PDZ canonical binding groove in accommodating different binding partners. Second, in addition to the canonical groove, other regions of the PDZ domain can also contribute to binding target proteins. This is well demonstrated in the α7 nAChR-PICK1 complex structure (Figure 6), where the MX helix and flexible loop of the α7 nAChR ICD interact with residues outside the canonical groove of the PICK1 PDZ domain. Third, PDZ-targeted proteins, such as the α7 nAChR ICD, must also possess sufficient plasticity to accommodate PDZ binding. The PICK1 PDZ domain would not fit well into a gap between two adjacent subunits of the α7 nAChR ICD if the ICD were not flexible.^16^ The α7 nAChR-PICK1 structure (Figure 6 and Supporting Information, Figure S6) demonstrates that flexible loops are as important as structured elements in the formation of a protein complex. Secondary structure analysis of the interface regions of protein complexes in the Protein Data Bank (PDB)^52^ shows an approximately equal proportion of helical, strand, and coil residues at the complex interfaces. Thus, it is conceivable that more PDZ complexes involving helical regions of target partners, like α7 nAChR-PICK1 or other unconventional binding motifs,^53^ will be identified in future studies.

Our structural study focuses on the PDZ domain of PICK1, excluding its BAR domain. Unlike the well-studied PDZ domain, the BAR domain remains structurally less explored. Currently, no experimental PICK1 structure exists encompassing both the PDZ and BAR domains, or even the BAR domain alone. Given our complex structure, can we infer that α7 nAChR binds to PICK1 in the presence of both domains? Computational PDZ-BAR models propose that a flexible linker of ∼40 amino acids between these two domains may provide the necessary plasticity to adapt to the structural requirements of different target receptors.^54,55^ Furthermore, binding of the ligand to the PDZ domain induces an intramolecular rearrangement, disrupting interactions between the PDZ-BAR domains. This rearrangement exposes the BAR domain, allowing it to bind to the membrane and facilitating PICK1’s association with target proteins.^56^ As shown in our α7 nAChR-PICK1 structure (Figure 6), the C-terminus of the PICK1 PDZ extends outside the α7 nAChR ICD, creating ample space and conformational freedom for the BAR domain to interact with the membrane.^56^ Intriguingly, previous X-ray scattering studies have suggested a banana-shaped antiparallel dimer of PICK1, with PDZ domains at the ends of the dimer.^57,58^ We hypothesize that a dimerized PICK1 is more likely to bind two individual receptors rather than a single α7 nAChR. This is due to the large radius of gyration (∼60 Å) of a PICK1 dimer,^57^ which significantly exceeds the gap between two adjacent subunits of the ICD.^16,40^ A future structural study involving α7 nAChR with full-length PICK1, encompassing both the PDZ and BAR domains, may provide additional insights into the interplay between these two proteins.

Clustering of neurotransmitter receptors within specific membrane regions is crucial for the normal function of receptors. While PICK1 typically promotes the clustering of target receptors,^8,10−12^ it does so differently with α7 nAChR as it reduces α7 nAChR clustering on the surface of GABAergic hippocampal interneurons.^14^ The reasons behind this reduced clustering could include PICK1-induced dispersion of α7 nAChR clusters, PICK1-promoted receptor internalization, or dynamic interactions between PICK1 and α7 nAChR in a “kiss and run” manner, preventing PICK1 from serving as a static anchor protein for α7 nAChR.^14^ However, none of these possibilities have been definitively proven through experimental evidence. Our structural model of the α7 nAChR-PICK1 complex (Figure 6) reveals intricate interweaving of these two proteins. Intuitively, based on this model, one would expect stronger PDZ domain binding in α7 nAChR-PICK1 complexes compared to those involving only a few C-terminal residues. Indeed, α7 nAChR binds to the PDZ domain of PICK1 with an affinity of ∼3 μM,^15,16^ which significantly surpasses the affinities observed for PDZ1 (∼100 μM) and PDZ1–2 (12 μM), and is comparable to the affinity of PDZ-95 (1.2 μM) for binding iGluRs.^59^ PDZ-95, a key scaffolding protein, plays a crucial role in anchoring iGluRs and other receptors to the cell surface membranes.^60−65^ Interestingly, despite similar binding affinities, PICK1 fails to stabilize α7 nAChR clusters in the same manner that PSD-95 does for iGluRs.^66−70^ The underlying reasons for this discrepancy remain an open question for future research.^71−74^

## Materials and Methods

### Protein Expression and Purification and Sample Preparations

Full-length WT and mutated human α7 nAChR,^15,75^ the α7 nAChR TMD + ICD,^16,40^ and the PDZ domain of human PICK1 containing a polyhistidine-tag and/or a strep tag were expressed in *Escherichia coli* Rosetta 2(DE3) pLysS (Novagen) using LB broth supplemented with 0.5 M sorbitol and 10 mM MgCl_2_ or M9 minimal media at 15 °C for ∼16 h (full length α7 nAChR and PICK1) or ∼72 h (TMD + ICD). The α7 nAChR deletion mutants Δ408–432, Δ419–423, and Δ428–432 were generated from the full-length WT human α7 nAChR expression plasmid using overlapping polymerase chain reaction (PCR) with primers flanking the deleted region (Supporting Information, Table S3) and Q5 high-fidelity master mix (New England Biolabs). The purified PCR products were treated with DpnI (New England Biolabs) to remove the WT plasmid and directly transformed to *E. coli* for amplification. All of the constructs were confirmed by sequence analysis before expression. The α7 purification followed previously reported protocols.^16,75^ Briefly, the proteins were purified with the appropriate resin, either NiNTA (HisTrap HP, Cytiva, UK or SuperNiNTA, Protein Ark, UK) or StrepTrap XT (Cytiva, UK). The pentamer fraction of α7 nAChR was isolated by size exclusion chromatography (SEC). PICK1 was purified using the published protocol^24^ with some modifications. Briefly, harvested cells were resuspended and lysed in 25 mM Tris, pH 7.4, 125 mM NaCl, 1 mM DTT, and HALT protease using a Microfluidics M-110Y microfluidizer. The lysate was mixed with 1% (w/v) lauryldimethylamine *N*-oxide (LDAO, MilliporeSigma), incubated by inversion at 4 °C for 1 h, and centrifuged at 30,000 g for 1 h in an SS-34 rotor. The supernatant was purified using a 5 mL StrepTrap XT column in the lysis buffer (without HALT) with 0.15% LDAO, and the protein was eluted with 50 mM biotin. For NMR experiments, PICK1 was cleaved with TEV protease, and the cleaved monomeric fraction was obtained using a Superdex 200 10/300 SEC column (Cytiva, UK) equilibrated with 25 mM Tris, pH 7.4, 0.02% LDAO, and 125 mM NaCl. Protein purity was confirmed by SDS-PAGE. MTSL labeling of cysteine residues in the PICK1 PDZ domain was achieved by the protocol published previously.^45,76^ About 15- to 25-fold molar excess of the nitroxide spin label MTSL (Toronto Research Chemicals) was added to the protein samples for ∼2 h at room temperature followed by overnight incubation at 4 °C. Free MTSL was removed through dialysis and subsequent SEC. The α7 nAChR TMD + ICD or/and the PICK1 PDZ domain in LDAO micelles were used for NMR. For detecting α7 NMR signals, a typical sample contained 150–200 μM ^15^N-labeled α7 TMD + ICD in the absence or presence of unlabeled PICK1, 0.5–1% LDAO, 25 mM NaCl, and 5 mM sodium acetate at pH 5. For detecting PICK1 NMR signals, the samples contained 50–100 μM ^15^N-labeled PICK1, without or with the unlabeled α7 nAChR TMD + ICD, in 25 mM Tris, pH 7.4, 0.02–0.03% LDAO, and 125 mM NaCl. 5% D_2_O and 20 μM DSS were added to the samples for deuterium lock and chemical shift calibration, respectively.

### Pulldown Using Purified Proteins

His-tag-containing WT α7 nAChR and mutants α7Δ419–423, α7Δ428–432, and α7Δ408–432 as well as the strep-tag-containing PICK1 PDZ constructs were expressed and purified for pulldown experiments. The final purified proteins were collected individually from SEC using a Superose 6 Increase 15/300 GL column with 10 mM phosphate buffer at pH 7.8, 150 mM NaCl, and 0.05% LDAO. Each α7 sample (0.15 mg/mL, ∼3 μM) was mixed with the PICK1 PDZ (0.15 mg/mL, ∼10 μM) in a molar ratio of 1:5 in an Eppendorf tube and incubated/shaken for 1 h at 4 °C. Then, super Ni-NTA affinity resin (30 μL) was added to each mixed sample, followed by overnight incubation. Next morning, the samples were centrifuged at 1000 g for 2 min at 4 °C. The supernatant was removed, and the resin was washed two times using 360 μL of SEC buffer containing 50 mM imidazole. The proteins were eluted with 30 μL of 500 mM imidazole. The control group for pulldown experiments used only PICK1 and Ni-NTA resin without the α7 protein to confirm that there was no nonspecific PICK1 binding to the resin under the same pulldown protocol. The final pulldown samples, including the control, along with the original input proteins, were loaded to 15% SDS-PAGE gel to demonstrate pulldown results. The band intensities (areas) of α7 and PICK1 PDZ samples were measured by using Adobe Photoshop 2024.

### NMR Spectroscopy

NMR spectra were acquired on a Bruker AVANCE 800 MHz spectrometer equipped with a triple-resonance inverse-detection TCI cryoprobe (Bruker Instruments) at different temperatures (288, 308, and 318 K). TopSpin 3.2 (Bruker), NMRPipe 3.19,^77^ and NMRFAM-SPARKY 1.470^78^ powered by Sparky 3.190^79^ were used for NMR data acquisition, processing, and spectral analysis, respectively. The most relevant data acquisition parameters along with a list of NMR experiments are summarized in Supporting Information, Table S4. The ^1^H chemical shifts were referenced to the DSS resonance at 0 ppm, and the ^15^N chemical shifts were referenced indirectly. The previously reported backbone chemical shift assignments of the α7 nAChR TMD + ICD^16,40^ and the PDZ domain of PICK1^24^ were used for the current NMR studies. 2D ^1^H–^15^N transverse relaxation optimized spectroscopy (TROSY)–HSQC CSP, PRE, and STD NMR experiments^43,44^ were used to determine the binding interface between the α7 nAChR TMD + ICD and the PDZ domain of PICK1. STD spectra were collected in an interleaved fashion with off- and on-resonance saturation of ^1^HN peak (∼11.6 ppm) of I33 for 0.5 s saturation and a recycle time of 1.5 s. The off-resonance frequency was set at 20 ppm, which is far from the ^1^H frequencies of both the ^15^N-labeled α7 nAChR TMD + ICD and the unlabeled PICK1 PDZ domain. Selective saturation was achieved using an IBURP2 pulse train (50 ms Gaus1.1000-shaped with an interpulse delay of 4 μs). 1D ^1^H STD spectra were recorded with a spectral window of 16 ppm and 16,384 data points. 2D ^1^H–^15^N TROSY–HSQC saturation transfer spectra had windows of 13 × 23 ppm and 2048 × 160 data points in the ^1^H and ^15^N dimension, respectively. Decreases in the α7 nAChR TMD + ICD cross-peak intensity upon saturation of PICK1 I33 ^1^HN were analyzed and used to determine direct interactions between residues of the two proteins. The ^1^H–^15^N TROSY–HSQC CSP NMR experiments were performed with the ^15^N-labeled α7 nAChR TMD + ICD in the presence of the unlabeled PICK1 PDZ domain (0, 65, and 210 μM) or the ^15^N-labeled PICK1 PDZ domain in the absence or presence of the unlabeled α7 nAChR TMD + ICD. Spectral windows in the ^1^H and ^15^N dimension were 13 × 23 ppm and 2048 × 160 data points, respectively, for the ^15^N-labeled α7 TMD + ICD and 16 × 30 ppm and 2048 × 128 data points, respectively, for the ^15^N-labeled PICK1. 2D PRE NMR experiments were performed with the isotope-labeled α7 nAChR TMD + ICD in the presence of the PICK1 PDZ domain without isotope labeling but with the paramagnetic probe MTSL at residues C44 and C46. ^1^H–^15^N TROSY-HSQC spectra in the paramagnetic (*I*) and diamagnetic (*I*_0_) conditions were acquired with spectral windows of 13 × 23 ppm and 2048 × 176 data points in the ^1^H_N_ and ^15^N dimensions, respectively. The diamagnetic condition was introduced by ascorbic acid (2.5 mM). Distance restraints were derived based on the Solomon and Bloembergen equation^80^ and used along with other structural restraints for structure calculations.^16^

### Modeling of the α7 nAChR-PICK1 Complex

HADDOCK2.4^49,50^ was used to generate protein complex models based on structure restraints derived from NMR experiments. The previously published NMR structures of α7 nAChR TMD + ICD (PDBID: 7RPM)^16^ and the PICK1 PDZ domain (PDBID: 2LUI)^24^ were used for initiating complex modeling. Note that the DTA C-terminal residues in the original structure of the PICK1 PDZ domain (PDBID: 2LUI) were removed in the complex modeling. The standard HADDOCK protocols were followed with modifications, including increasing the number of trials for rigid body minimization from 5 to 10 and adding experimental restraints between α7 nAChR and PICK1. Chiron^81^ was used to minimize steric clashes. The final selection of the complex structure (PDBDEV_00000230) was based on the best HADDOCK score, Z-score, and low restraints violation energy. The quality of the final structures was evaluated using MolProbity^82^ and Phenix^83^ 1.19 (Supporting Information, Table S2). Visual molecular dynamics^84^ was used for visualization and analysis.

## Data Availability

The atomic coordinates and structure restraints for the representative structure of the α7 nAChR TMD + ICD in complex with the PICK1 PDZ domain have been deposited in the PDB-Dev (https://pdb-dev.wwpdb.org/) with the accession code PDBDEV_00000230. The source data underlying Figures 3b, 4c, and 5b are provided as a Source Data file. The chemical shift values have been deposited in the Biological Magnetic Resonance Data Bank (BMRB) with accession numbers 52246, 52247, 52248, and 52249. Other data that support the findings of this study are available upon reasonable request to the corresponding author.

## Supporting Information Available

Impact of the α7nAChR MA helix deletion to binding PICK1; overlay of 2D ^1^H-^15^N TROSY-HSQC NMR spectra of α7nAChR TMD+ICD in the presence of different concentrations of PICK1 PDZ domain; overlay of 2D (STD) NMR spectra of α7nAChR TMD+ICD; inter-protein paramagnetic relaxation enhancement; FSC231 inhibition of PICK1 pulldown by α7nAChR; representative interactions between α7nAChR ICD; impact of mutations in the MA helix of α7nAChR to binding the PICK1 PDZ domain; HADDOCK output parameters; quality evaluation of the α7nAChR + PICK1 complex mode; oligos for α7nAChR mutagenesis; and NMR samples, experiments, and most relevant acquisition parameters (PDF)
