# Supplementary material for: Unconventional PDZ Recognition Revealed in α7 nAChR-PICK1 Complexes
Source: ACS Chem Neurosci. 2024 May 1;15(10):2070–9. doi: 10.1021/acschemneuro.4c00138 (PMC11099923; doi:10.1021/acschemneuro.4c00138)
Supplement: Supplementary file 1 — cn4c00138_si_001.pdf [file cn4c00138_si_001.pdf]

Supplementary Information

Unconventional PDZ Recognition Revealed in the α7nAChR-PICK1 Complex

Vasyl Bondarenko[1], Qiang Chen[1], Tommy S. Tillman[1], Yan Xu[1,2,3,4], Pei Tang[1,3,5*]

[1]Depatment of Anesthesiology and Perioperative Medicine, University of Pittsburgh, Pittsburgh, PA 15260, USA

[2]Department of Structural Biology, University of Pittsburgh, Pittsburgh, PA 15260, USA

[3]Department of Pharmacology and Chemical Biology, University of Pittsburgh, Pittsburgh, PA 15260, USA

[4]Department of Physics and Astronomy, University of Pittsburgh, Pittsburgh, PA 15260, USA;

[5]Department of Computational and Systems Biology, University of Pittsburgh, Pittsburgh, PA 15260, USA

[*]Correspondence and requests for materials should be addressed to P.T. (email: ptang@pitt.edu)

There are seven Supporting Figures and four Supporting Tables

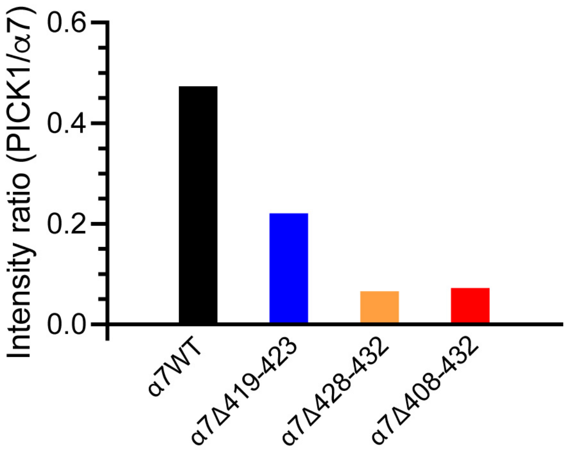

	Intensity ratio (PICK1/ α7)	Normalized Intensity ratio	% decrease
α7WT	0.473	1	
α7Δ419-423	0.221	0.467	53.3%
α7Δ428-432	0.067	0.142	85.8%
α7Δ408-432	0.072	0.152	84.8%

Figure S1. Impact of the α7nAChR MA helix deletion to binding PICK1. Intensity ratios (PICK1/α7nAChR) are obtained from measuring individual band intensities in each lane of the SDS-page resulted from pulldown experiments (Fig. 2). Relative to the full length α7nAChR (α7WT), deletion of MA residues reduced PICK1 pulldown. α7Δ419-423 in the middle of the MA helix decreased PICK1 pulldown by ~53%. α7Δ428-432 toward top of the MA helix decreased PICK1 pulldown by ~86%. Interestingly, α7Δ408-432, a much more extended deletion, resulted in almost the same decrease (~85%) as α7Δ428-432. The results suggest that the upper part of the MA helix plays a more important role in binding PICK1 than the lower part of the MA helix.

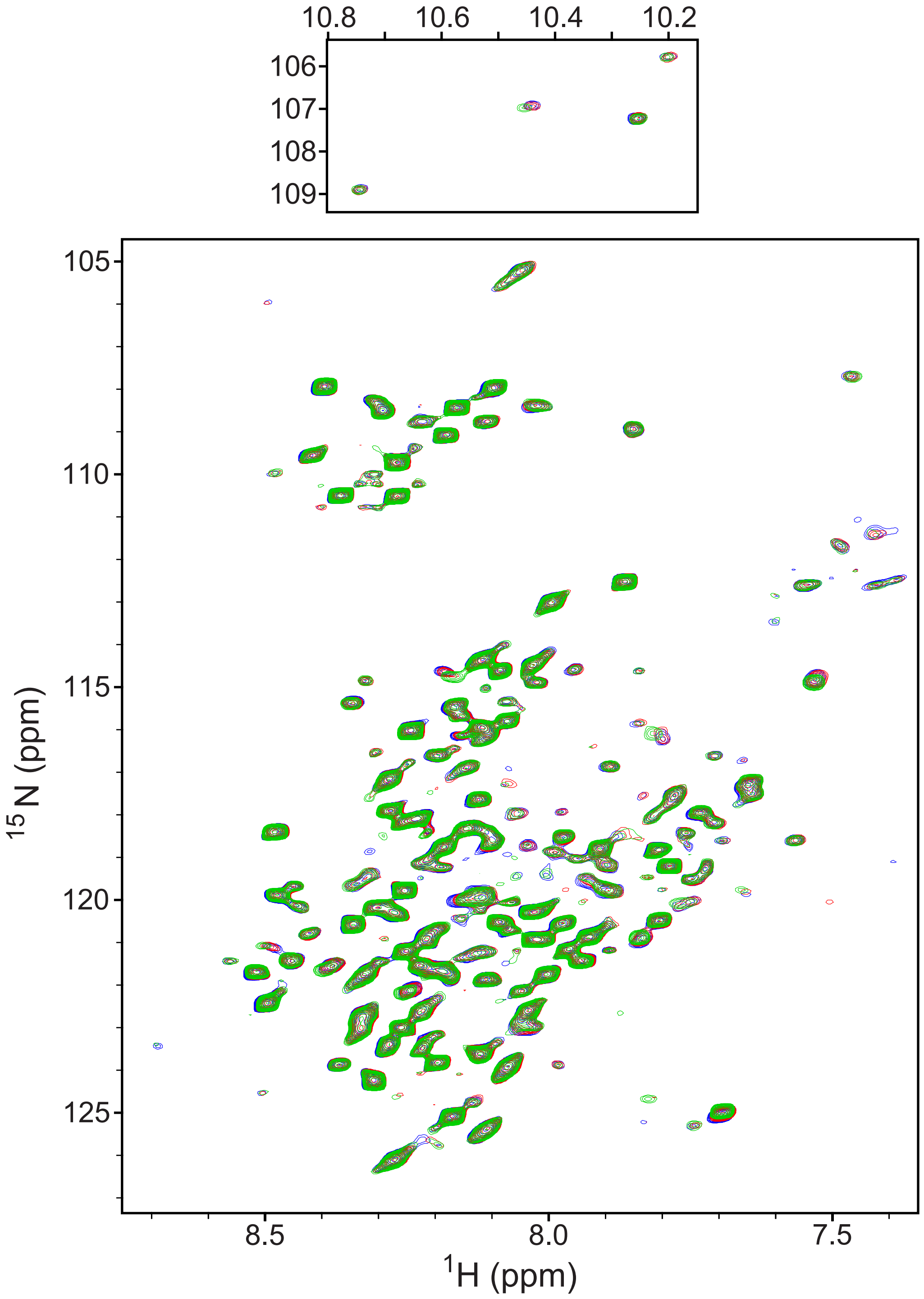

Figure S2. Overlay of 2D ^{1}H-^{15}N TROSY-HSQC NMR spectra of the α7nAChR TMD+ICD in the presence of different concentrations of the PICK1 PDZ domain, 0 (blue), 65 µM (red), and 210 µM (green). The top spectra show the region of tryptophan sidechain indoles. These spectra were acquired at 800 MHz NMR spectrometer at 45 ºC.

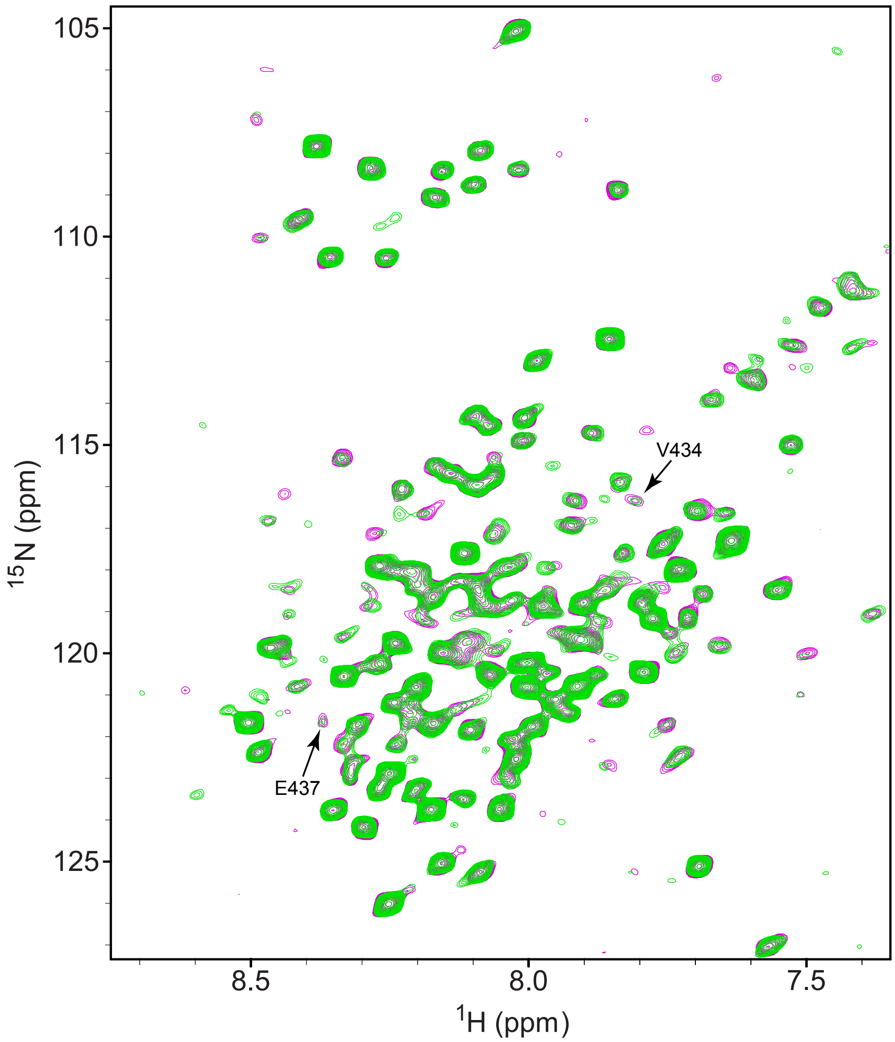

Figure S3. Overlay of 2D saturation transfer (STD) NMR spectra of the α7nAChR TMD+ICD without (purple) and with (green) saturation of the PICK1 I33 amide proton (~11.6 ppm). STD spectra were collected in an interleaved fashion with off- and on-resonance saturation of ^{1}HN peak (~11.6 ppm) of I33 for 0.5s saturation and a recycle time of 1.5s. The off-resonance frequency was set at 20 ppm, which is far away from the ^{1}H frequencies of both the ^{15}N-labeled α7nAChR TMD+ICD and the unlabeled PICK1 PDZ domain. The selective saturation was achieved using an IBURP2 pulse train (50 ms Gaus1.1000-shaped with an inter-pulse delay of 4 µs). The spectra were acquired at 800 MHz NMR spectrometer at 45 ºC.

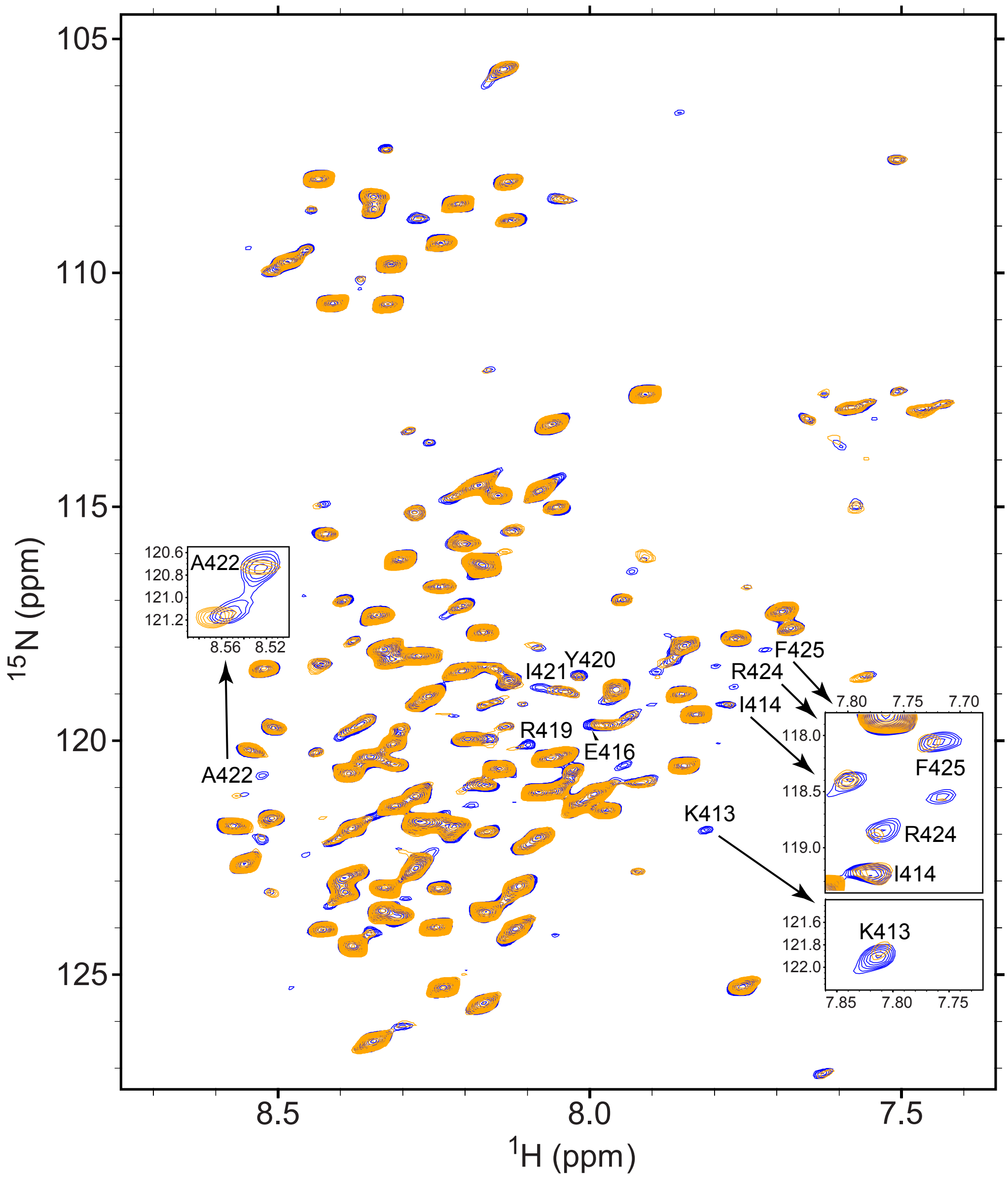

Figure S4. Inter-protein paramagnetic relaxation enhancement (PRE) NMR. 2D ^{1}H-^{15}N TROSY-HSQC NMR spectra of the α7nAChR TMD+ICD were collected in the presence of PICK1 PDZ domain in the paramagnetic (**orange**) and diamagnetic (**blue**) conditions, respectively. The paramagnetic condition resulted from the MTSL labeling at residues C44 and C46 of the PDZ. The diamagnetic condition was introduced by ascorbic acid (2.5 mM). Several inserts are provided to assist visualization of signal changes under the paramagnetic and diamagnetic conditions. These spectra were acquired at 800 MHz NMR spectrometer at 35 ºC.

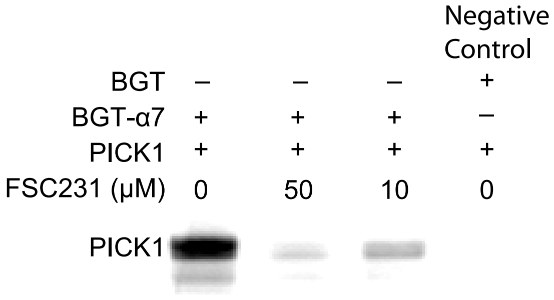

Figure S5. FSC231 inhibition of PICK1 pulldown by α7nAChR. (**a**) PICK1 was pulled down by α7nAChR conjugated bungarotoxin resin (BGT-α7), but not resin alone (BGT). (**b**) The PICK1 inhibitor FSC231 inhibited PICK1 binding to BGT-α7 in a concentration dependent manner. All samples were washed five times in PBS with 0.1% Triton X100, resolved by SDS-PAGE, and stained with Sypro Ruby.

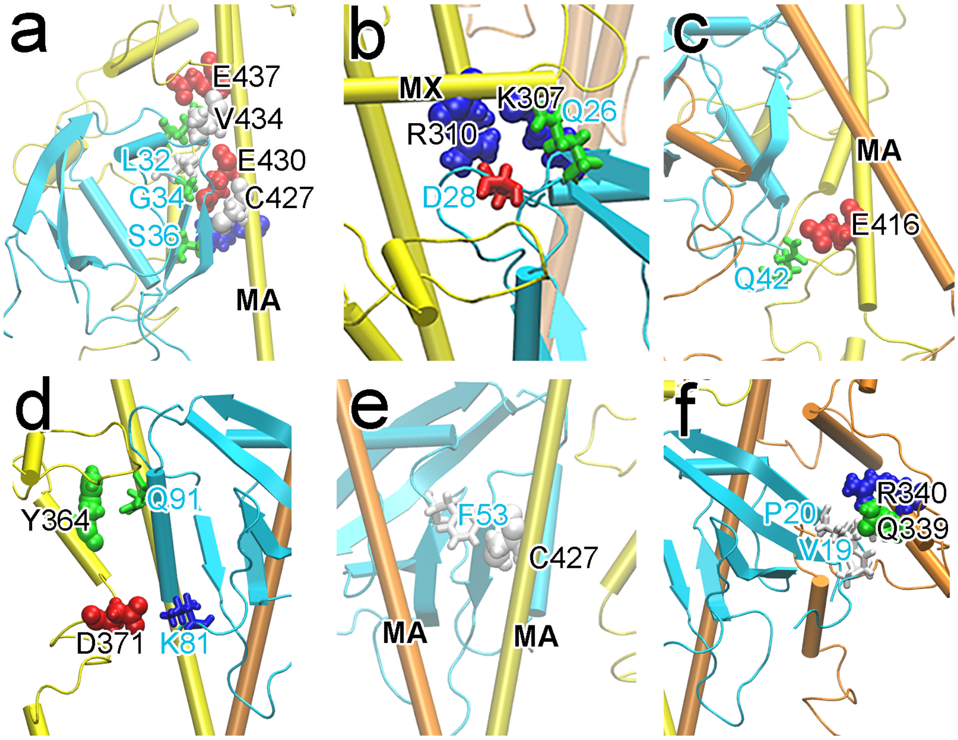

Figure S6. Representative interactions between α7nAChR ICD (colored in yellow or orange, residues are presented in VDW and label in black) **and PICK1 PDZ** (colored in cyan, residues are presented in licorice and labeled in cyan). (**a**) Interactions between the α7nAChR MA helix and PICK1 βB that lines the canonical binding grove of the PDZ domain. (**b**) Interactions between the α7nAChR MX helix and loop residues prior the PICK1 βB. (**c**) α7nAChR ICD residues at the lower MA helix (E416) contacts with the PICK1 loop residue Q42. (**d**) α7nAChR residues in the ICD flexible loop interact with PICK1 residues prior (K81) and on (Q91) αB helix. Note the salt bridge between D371 and K81. (**e**) and (**f**) Residues of the PICK1 PDZ domain interact with residues not only on the principal side (yellow) but also complementary side (orange) of α7nAChR subunits.

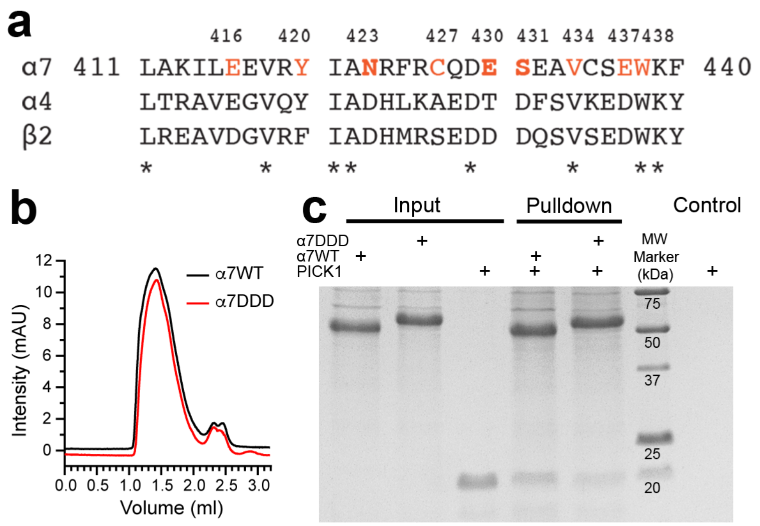

Figure S7. Impact of mutations in the MA helix of α7nAChR to binding the PICK1 PDZ domain. (**a**) Sequence alignment of MA helices of the human α7, α4, and β2 nAChRs. The α7 residues colored in orange are closely interacting with the PDZ domain in the α7nAChR-PICK1 complex structural model. To validate the complex structure and assess how mutations in the α7 MA helix influence the α7nAChR-PICK1 complex formation, we designed an α7 mutant of three mutations (N423D, E430D and S431D), named α7DDD, to match these three α7 residues with the equivalent residues in α4 or/and β2, because neither α4 nor β2 was found binding PICK1 previously.[1] (**b**) Overlay of size exclusive chromatography (SEC) profiles of the purified wild type (α7WT, black) and mutant (α7DDD, red) α7nAChR. The same SEC profile and peak position (~1.4 ml) of α7WT and α7DDD suggests that α7DDD retains the pentameric assembly as α7WT. The SEC was performed using a Superdex 200 Increase 3.2/300 column with 10 mM phosphate buffer at pH 7.8, 150 mM NaCl and 0.05% LDAO. (**c**) 15% SDS-PAGE gel showing pulldown of the PICK1 PDZ domain by α7WT and α7DDD with their his-tags as baits. Comparing to α7WT, α7DDD shows a ~50% decrease in pulldown of PICK1 based on band intensity ratios of PICK1/α7WT vs. PICK1/α7DDD. The slower migration of α7DDD in the SDS-PAGE results from an increase of the net negative charge in α7DDD as reported previously.[2] The experiment was carried out following the protocol described in the Method section.

1. Baer, K.; Burli, T.; Huh, K. H.; Wiesner, A.; Erb-Vogtli, S.; Gockeritz-Dujmovic, D.; Moransard, M.; Nishimune, A.; Rees, M. I.; Henley, J. M.; Fritschy, J. M.; Fuhrer, C., PICK1 interacts with alpha7 neuronal nicotinic acetylcholine receptors and controls their clustering. *Mol Cell Neurosci* 2007, 35, 339-55.

2. Shi, Y.; Mowery, R. A.; Ashley, J.; Hentz, M.; Ramirez, A. J.; Bilgicer, B.; Slunt-Brown, H.; Borchelt, D. R.; Shaw, B. F., Abnormal SDS-PAGE migration of cytosolic proteins can identify domains and mechanisms that control surfactant binding. *Protein Sci* 2012, 21, 1197-209.

Table S1. HADDOCK output parameters

**Cluster 1**	
HADDOCK score	-159.5 ± 8.8
Cluster size	135
RMSD from the overall lowest-energy structure	0.6 ± 0.4
Van der Waals energy	-88.6 ± 4.4
Electrostatic energy	-290.1 ± 33.9
Desolvation energy	-20.5 ± 1.2
Restraints violation energy	76.8 ± 26.9
Buried Surface Area	3154.1 ± 207.2
Z-Score	-1.6

Table S2. Quality evaluation of the α7nAChR + PICK1 complex model*

**Deviations from idealized geometry**	
Bond lengths (Å)	0.0034
Bond angles (º)	0.89
Rotamer Outliers (%)	0.08
Clash score	1.7
MolProbity score	0.92
**Ramachandran plot**	
favored (%)	98.14
outliers (%)	0.00

*The reported parameters were resulted from Phenix.

Table S3. Oligos for α7nAChR Mutagenesis

α7 Δ408-432 reverse	CAGACCGCCCCCTCGGGGGGTTG
α7 Δ408-432 forward	CCGAGGGGGCGGTCTGCAGCGAGTG
α7 Δ419-423 reverse	GGAAGCGGACCTCCTCCAGGATCTTG
α7 Δ419-423 forward	AGGAGGTCCGCTTCCGCTGCCAG
α7 Δ428-432 reverse	CAGACCGCGCAGCGGAAGCGGTTGG
α7 Δ428-432 forward	TCCGCTGCGCGGTCTGCAGCGAGTG

Supplementary Table 4. NMR samples, experiments, and most relevant acquisition parameters

**NMR experiment**	**Spectral width (ppm)**	**Data points in time domain**	**Number of scans**	**Data collection time**	**Other notes**
Titration NMR experiments to determine the α7nAChR TMD+ICD – PICK1 PDZ domain interaction site (determination of changes in α7 TMD+ICD backbone $^{1}H_{N}$ and ^{15}N chemical shifts and intensity of ^{1}H-^{15}N cross-peaks induced by PICK1)					
**Sample 1-3**: 0.2 mM ^{15}N-labeled α7nAChR TMD+ICD without/with PICK1, 5 mM sodium acetate pH5, 20 mM (0.5%) LDAO, 25 mM NaCl; magnetic field: 18.8 T; temperature: 318 K					
2D ^{1}H-^{15}N TROSY-HSQC	13×23 ppm ($^{1}H_{N}$×^{15}N)	1024×160 ($^{1}H_{N}$×^{15}N)	48	3 h	0 mM PICK1
2D ^{1}H-^{15}N TROSY-HSQC	13×23 ppm ($^{1}H_{N}$×^{15}N)	1024×160 ($^{1}H_{N}$×^{15}N)	64	4 h	0.065 mM PICK1
2D ^{1}H-^{15}N TROSY-HSQC	13×23 ppm ($^{1}H_{N}$×^{15}N)	1024×160 ($^{1}H_{N}$×^{15}N)	72	4.5 h	0.210 mM PICK1
Paramagnetic Relaxation Enhancement (PRE) NMR experiments to determine the α7nAChR TMD+ICD – PICK1 PDZ domain interaction site					
**Sample 4**: 0.2 mM ^{15}N-labeled α7nAChR TMD+ICD with 0.25 mM MTSL-labeled PICK1, 5 mM sodium acetate pH5, 20 mM (0.5%) LDAO, 25 mM NaCl; magnetic field: 18.8 T; temperature: 308 K					
2D ^{1}H-^{15}N TROSY-HSQC	13×23 ppm ($^{1}H_{N}$×^{15}N)	2048×176 ($^{1}H_{N}$×^{15}N)	64	4 h	0.25 mM MTSL-PICK1; 0 and 2.5 mM ascorbic acid
1D and 2D Saturation Transfer Difference (STD) NMR experiments to determine the α7nAChR TMD+ICD – PICK1 PDZ domain interaction site (determination of α7 TMD+ICD residues in direct interaction with PICK1 I33)					
**Sample 5**: 0.2 mM ^{15}N-labeled α7nAChR TMD+ICD with 0.25 mM PICK1, 5 mM sodium acetate pH5, 35 mM (0.8%) LDAO, 25 mM NaCl; magnetic field: 18.8 T; temperature: 318 K (saturation of PICK1 I33 H_{N} at 11.6 ppm for 0.5 s; relaxation delay: 1.5 s)					
1D STD	20 ppm	16384	32	3 min	0.25 mM PICK1
2D ^{1}H-^{15}N STD TROSY-HSQC	13×23 ppm ($^{1}H_{N}$×^{15}N)	2048×160 ($^{1}H_{N}$×^{15}N)	48	7 h	0.25 mM PICK1
Titration NMR experiments to determine the α7nAChR TMD+ICD – PICK1 PDZ domain interaction site (determination of changes in PICK1 backbone $^{1}H_{N}$ and ^{15}N chemical shifts and intensity of ^{1}H-^{15}N cross-peaks induced by α7 TMD+ICD)					
**Sample 6-7 (cleaved)**: 0.07 mM ^{15}N-labeled PICK1 without/with 0.01 mM α7nAChR TMD+ICD, 25 mM Tris pH7.4, 1.3 mM (0.03%) LDAO, 25 mM NaCl; magnetic field: 18.8 T; temperature: 288 K					
2D ^{1}H-^{15}N HSQC	16×30 ppm ($^{1}H_{N}$×^{15}N)	2048×128 ($^{1}H_{N}$×^{15}N)	48	2 h	0 mM α7 TMD+ICD
2D ^{1}H-^{15}N HSQC	16×30 ppm ($^{1}H_{N}$×^{15}N)	2048×128 ($^{1}H_{N}$×^{15}N)	96	4 h	0.01 mM α7 TMD+ICD

^1H_N and ^{15}N PICK1 PDZ domain chemical shift assignment					
**Sample 8 (cleaved)**: 0.1 mM ^{15}N-labeled PICK1, 25 mM Tris pH7.4, 1 mM (0.02%) LDAO, 125 mM NaCl, 1 mM DTT; magnetic field: 16.4 T; temperature: 278 K					
2D ^1H-^{15}N HSQC	15×30 ppm (^1H_N×^{15}N)	2048×160 (^1H_N×^{15}N)	48	2.5 h	
3D ^{15}N-edited NOESY-HSQC	15×30×15ppm (^1H_N×^{15}N×^1H)	2048×48×128 (^1H_N×^{15}N×^1H)	8	14 h	NOE mixing time: 200 ms
Examination of the PICK1 PDZ domain quality before TEV cleavage					
**Sample 9-10 (uncleaved)**: 0.1 mM ^{15}N-labeled PICK1, 25 mM Tris pH7.4, 1.3 mM (0.03%) LDAO, 125 mM NaCl, 1 mM DTT; magnetic field: 16.4 T; temperature: 278, 288 and 298 K					
2D ^1H-^{15}N HSQC	15×30 ppm (^1H_N×^{15}N)	2048×128 (^1H_N×^{15}N)	48	2 h	278 K
2D ^1H-^{15}N HSQC	15×30 ppm (^1H_N×^{15}N)	2048×128 (^1H_N×^{15}N)	48	2 h	288 K
2D ^1H-^{15}N HSQC	15×30 ppm (^1H_N×^{15}N)	2048×128 (^1H_N×^{15}N)	48	2 h	298 K

*A recycle delay (D1) of 1 s was used in all NMR experiments, except 1D and 2D ^1H-^{15}N STD experiments (1.5 s)

All samples contain 20 μM DSS for the reference of the 0 ppm ^1H chemical shift.
